# Biological Activities of Lichen-Derived Monoaromatic Compounds

**DOI:** 10.3390/molecules27092871

**Published:** 2022-04-30

**Authors:** Thanh-Hung Do, Thuc-Huy Duong, Huy Truong Nguyen, Thi-Hien Nguyen, Jirapast Sichaem, Chuong Hoang Nguyen, Huu-Hung Nguyen, Nguyen Phuoc Long

**Affiliations:** 1 NTT Hi-Tech Institute, Nguyen Tat Thanh University, Ho Chi Minh City 700000, Vietnam; dthung@ntt.edu.vn; 2 Department of Chemistry, University of Education, 280 An Duong Vuong Street, District 5, Ho Chi Minh City 700000, Vietnam; nguyenhienhcmue@gmail.com; 3 Application in Pharmaceutical Sciences Research Group, Faculty of Pharmacy, Ton Duc Thang University, Ho Chi Minh City 700000, Vietnam; nguyentruonghuy@tdtu.edu.vn; 4 Research Unit in Natural Products Chemistry and Bioactivities, Faculty of Science and Technology, Thammasat University Lampang Campus, Lampang 52190, Thailand; jirapast@tu.ac.th; 5 University of Science, Vietnam National University Ho Chi Minh City, Ho Chi Minh City 700000, Vietnam; nhchuong@hcmus.edu.vn; 6 Faculty of Applied Technology, School of Engineering and Technology, Van Lang University, Ho Chi Minh City 700000, Vietnam; hung.nh@vlu.edu.vn; 7 Department of Pharmacology and PharmacoGenomics Research Center, Inje University College of Medicine, Busan 614-735, Korea

**Keywords:** lichen, *Parmotrema*, *Roccella*, monoaromatic compounds, antimicrobial activity, alpha-glucosidase inhibition

## Abstract

Lichen-derived monoaromatic compounds are bioactive compounds, associated with various pharmacological properties: antioxidant, antifungal, antiviral, cytotoxicity, and enzyme inhibition. However, little is known about data regarding alpha-glucosidase inhibition and antimicrobial activity. Very few compounds were reported to have these activities. In this paper, a series of monoaromatic compounds from a lichen source were isolated and structurally elucidated. They are 3,5-dihydroxybenzoic acid (**1**), 3,5-dihydroxybenzoate methyl (**2**), 3,5-dihydroxy-4-methylbenzoic acid (**3**), 3,5-dihydroxy-4-methoxylbenzoic acid (**4**), 3-hydroxyorcinol (**5**), atranol (**6**), and methyl hematommate (**7**). To obtain more derivatives, available compounds from the previous reports such as methyl β-orsellinate (**8**), methyl orsellinate (**9**), and D-montagnetol (**10**) were selected for bromination. Electrophilic bromination was applied to **8**–**10** using NaBr/H_2_O_2_ reagents to yield products methyl 5-bromo-β-orsellinate (**8a**), methyl 3,5-dibromo-orsellinate (**9a**), 3-bromo-D-montagnetol (**10a**), and 3,5-dibromo-D-montagnetol (**10b**). Compounds were evaluated for alpha-glucosidase inhibition and antimicrobial activity against antibiotic-resistant, pathogenic bacteria *Enterococcus faecium*, *Staphylococcus aureus*, and *Acinetobacter baumannii*. Compound **4** showed stronger alpha-glucosidase inhibition than others with an IC_50_ value of 24.0 µg/mL. Synthetic compound **9a** exhibited remarkable activity against *Staphylococcus aureus* with a MIC value of 4 µg/mL. Molecular docking studies were performed to confirm the consistency between in vitro and in silico studies.

## 1. Introduction

Lichens have produced hundreds of bioactive compounds with a range of skeletal forms [1,2,3]. They are associated with various pharmacological properties, being a blocker of UV radiation, antioxidant [4], antifungal [5], an inhibitor of cancer cells [6], antiviral, antimicrobial, and enzyme inhibitor [7,8]. Monocyclic aromatic compounds from lichen sources are believed to be bioactive sources. They showed potent cytotoxicity against cancer cell lines [9,10], alpha-glucosidase inhibitory [7,11], anti-inflammatory [6], anti-leishmanial [12], antifungal [13], and antimicrobial activities [6,14].

Lichen-derived monoaromatic compounds were divided into two sub-classes, orcinol, and β-orcinol, depending on the presence of a substituent at C-3. However, little is known about the antimicrobial and alpha-glucosidase inhibition of monoaromatic compounds and derivatives. As a few examples, orcinol, methyl orsellinate, ethyl orsellinate, and methyl β-orsellinate showed antimicrobial activities on various microorganisms with MIC values in the range of 30–500 µg/mL [6,14]. Regarding alpha-glucosidase inhibition, methyl hematommate [11] and cristiferide A [7] showed the potent activity while methyl orsellinate and methyl β-orsellinate are weak inhibitors [15,16]. Synthetic derivatives of monoaromatic compounds were produced mainly through esterification and etherification [15,16] but their alpha-glucosidase inhibition has not been studied.

**Figure 1:**
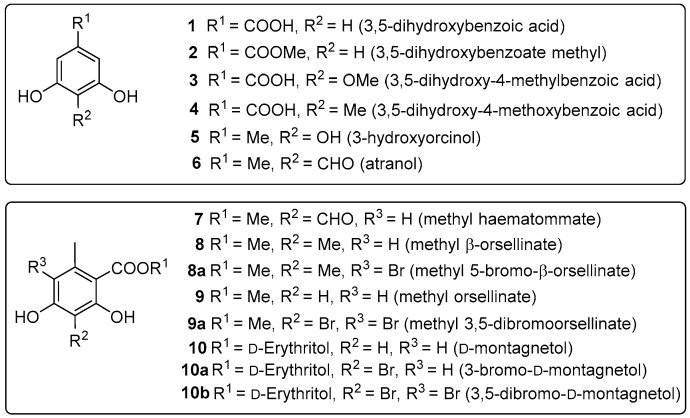
Chemical structures of **1**–**10**, **8a**, **9a**, **10a**, and **10b**.

**Figure 2:**
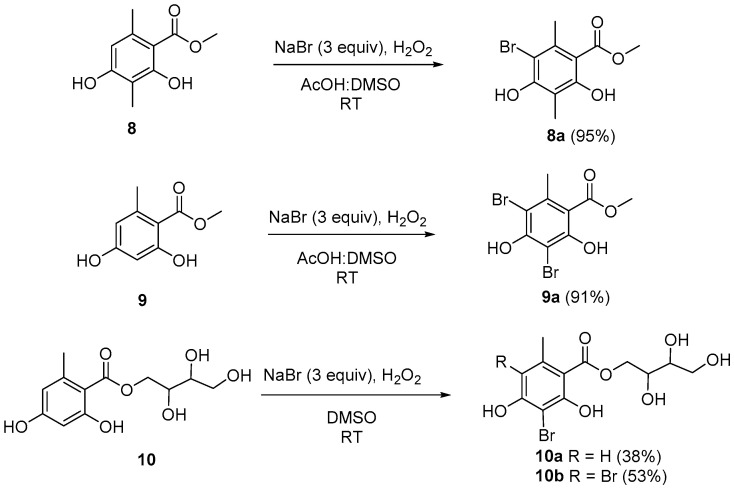
Pathway to preparation of **8a**, **9a**, **10a**, and **10b**.

In this study, seven natural monoaromatic compounds **1**–**7** were isolated from the lichen *Parmotrema* *cristiferum* (Figure 1). In addition, four synthetic compounds **8a**, **9a**, **10a**, and **10b** were prepared from the corresponding starting materials **8**–**10** (Figure 2). Their structures were elucidated by spectroscopic data analysis and comparison with literature data. Compounds were evaluated for alpha-glucosidase inhibition and antimicrobial activity against antibiotic-resistant, pathogenic bacteria *E. faecium, S. aureus*, and *A. baumannii*.

## 2. Results and Discussion

### 2.1. Phytochemical Identification of ***1***–***7***

From the lichen *Parmotrema* *cristiferum*, the EtOAc extract was prepared using maceration. While evaporating the solvent, the solid appeared and was collected. In this paper, compounds **1**–**7** were isolated from the solid part using multiple chromatographic methods. They were identified as 3,5-dihydroxybenzoic acid (**1**), 3,5-dihydroxybenzoate methyl (**2**), 3,5-dihydroxy-4-methylbenzoic acid (**3**), 3,5-dihydroxy-4-methoxylbenzoic acid (**4**), 3-hydroxyorcinol (**5**), atranol (**6**), and methyl hematommate (**7**). Their structures were elucidated by spectroscopic data analysis and comparison with literature data. Compounds **1**–**5** were reported from the *Parmotrema* genus for the first time. Methyl β-orsellinate (**8**) and methyl orsellinate (**9**) were isolated from the lichen *P. cristiferum* in the previous report [7]. Likewise, D-montagnetol (**10**) was available from the lichen *Roccella montagnei* [17].

### 2.2. Aromatic Bromination to Produce Compounds ***8a***, ***9a***, ***10a***, and ***10b***

**Figure 3:**
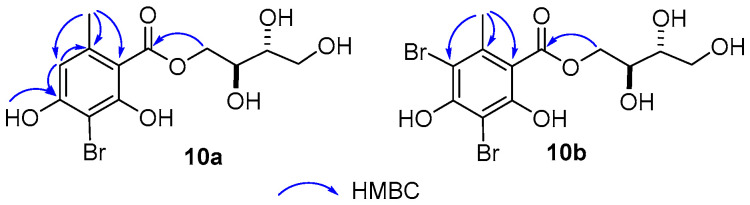
Key HMBC correlations of **10a** and **10b**.

Brominated substitution using sodium bromide and hydroperoxide was conducted on **8**, **9**, and **10** to provide products **8a**, **9a**, **10a**, and **10b**. This reaction was selected based on enhancing alpha-glucosidase inhibition of brominated derivatives from the previous reports. Particularly, the bromination of flavonoids, kamatakenin, and ayanin increased dramatically their alpha-glucosidase inhibition [18]. Further, brominated lichen metabolites showed more potent activity than their mother depsidones [19]. In contrast, esterification and etherification might decrease the activity [15,16]. Synthetic products were identified using HRESIMS, 1D-, and 2D-NMR (Figure 3).

### 2.3. Alpha-Glucosidase Inhibitory Activity of Compounds ***1***–***10***, ***8a***, ***9a***, ***10a***, and ***10b***

The in vitro alpha-glucosidase inhibitory activity of natural and synthetic compounds was evaluated (Table 1). Compounds **1**–**5**, **7**, **8a**, **9a**, **10a**, and **10b** displayed potent alpha-glucosidase inhibitory activity with IC_50_ values in the range of 24.0–171.1 µg/mL, compared with the 317 µg/mL of the acarbose positive control. Compounds **6** and **8**–**10** are inactive. Compounds **1**–**4** showed more potent activity than others, indicating the important role of 3,5-dihydroxy-1-carboxylbenzenoid ring in alpha-glucosidase inhibition. The 2,4-dihydroxy-1-carboxylbenzyl moiety of compounds **8**–**10** might decrease the activity. The results were similar to those reported by Lopes and co-workers (2008) [16]. Compound **7** was stronger than **6**, **8**, and **9**, proposing that the 3-CHO group exerted a significant effect on the activity. This was consistent with the data reported by Devi and co-workers (2021) [11].

As regards synthetic compounds, compounds **8a**, **9a**, **10a**, and **10b** showed improved activity than their mother compounds **8**, **9**, and **10**, indicating that the presence of the bromine atoms significantly increased the inhibition.

### 2.4. Antimicrobial Activity of Compounds ***1***–***10***, ***8a***, ***9a***, ***10a***, and ***10b***

Compounds **1**–**10**, **8a**, **9a**, **10a**, and **10b** were evaluated the antimicrobial activity against antibiotic-resistant, pathogenic bacteria *Enterococcus faecium, Staphylococcus aureus,* and *Acinetobacter baumannii* (Table 2). Compounds **1**–**7**, **10**, and **10b** are inactive against all tested strains. Compound **8** (methyl β-orsellinate) inhibited three selected strains with the inhibition zones of 18, 16, and 13 mm at the concentration of 50 µg/mL. Ingolfsdottir et al. (1985) reported that **8** could inhibit various microorganisms *S.* *aureus*, *Bacillus subtilis, Pseudomonas aeruginosa, Candida albicans, Escherichia coli*, and *Aspergillus niger* with MIC values in the range from 80–160 µg/mL [20]. This compound is stronger than methyl orsellinate (**9**). Interestingly, the synthetic products **8a** and **9a** obtained from **8** and **9** only showed the activity against *S.* *aureus*. Compound **9a** showed more potent activity than both **8** and **9**, indicating its selection against *S.* *aureus*. Its MIC value was 4 µg/mL compared to the positive control, kanamycin (MIC value of 4 µg/mL).

### 2.5. Molecular Docking Studies

**Figure 4:**
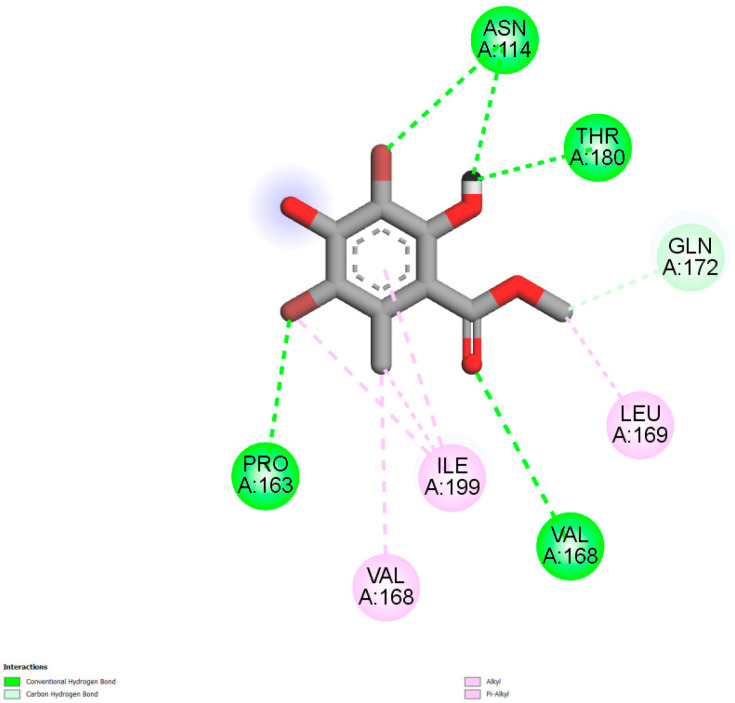
2D interaction diagram of ligand **9a**-1t2p.

The pose views showed that **9a** was strongly positioned in the 1t2p binding site by three h-bonds with Thr 180, Val 168, and Asn 114 and two halogen bonds with Asn 114 and Pro 163 (Figure 4), in which, the contribution to the binding site mechanism of residues such as Val 168, Thr 180, and Pro 163 has been demonstrated in the literature [21,22]. The free energy of this ligand was outstanding at −6.7 kcal/mol.

**Figure 5:**
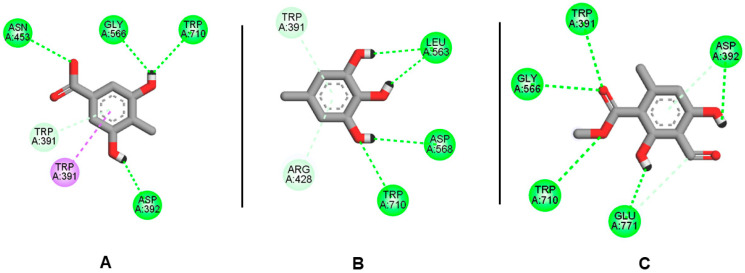
2D interaction diagram of ligand **3**-4j5t (**A**), **5**-4j5t (**B**), **7**-4j5t (**C**).

For 4j5t protein, compound **3** exhibited the lowest binding energy at −5.3 kcal/mol, followed by **7** and **5** at −4.8 and −4.3 kcal/mol respectively. The 3–4j5t complex was supported by four h-bonds with Trp 710, Gly 566, Asn 453, and Asp 392 (Figure 5). Similarly, Trp 710, Gly 566, and Asp 392 are key residues in the hydrolysis reaction at the active site of alpha-Glucosidase I. These residues appeared also in the binding mode of compounds **5** and **7**.

Generally, the estimated binding energies of the four ligands were consistent with the number of key interactions observed. However, with the central nucleus containing only one benzene nucleus and surrounded by polar functional groups (hydroxyl, ester, carboxylic acid), these four ligands lack the active support of hydrophobic interactions, which may explain the low affinity of those ligands to the target protein. The free energies and major residues of those complexes are presented in Table 3.

## 3. Materials and Methods

### 3.1. Source of the Lichen Material P. cristiferum

The thallus of lichen *P. cristiferum* was collected in Duc Trong district, Lam Dong province, Vietnam, in March 2020. The scientific name of the lichen was determined by Dr. Thi-Phi-Giao Vo, Faculty of Biology, Ho Chi Minh University of Science, National University—Ho Chi Minh City. A voucher specimen (UE-L006) was stored in the herbarium of the Department of Organic Chemistry, Ho Chi Minh University of Education.

### 3.2. Isolation of Compounds ***1***–***7*** from P. cristiferum

The clean, air-dried, and ground material (1.1 kg) was macerated in EtOAc at room temperature (10 L × 5 times, each time for 12 h) and the filtrated solution was evaporated under reduced pressure to produce the crude EtOAc extract (330 g). While the solvent was evaporated, the solid (**T**, 8.1 g) appeared and was separated by the Büchner funnel. This solid was dissolved in methanol, giving the solid and solution parts. The methanolic solution was applied to Sephadex LH-20 gel chromatography (Sigma Aldrich Co, St. Louis, MO, USA), and eluted with methanol to afford five fractions (T1-T5). The fraction T5 (2.1 g) was subjected to silica gel column chromatography (CC) using a mobile phase of *n*-hexane-EtOAc (stepwise, 5:1, 3:1, 1:1, *v*/*v*) to give 5 fractions (T1.1- T1.5). Fraction T1.1 (125 mg) was rechromatographed by silica gel CC with *n*-hexane-EtOAc: AcOH (5:1:0.02, *v*/*v*/*v*), afford compounds **6** (3 mg) and **7** (12 mg). Fraction T1.5 was applied to preparative thin-layer chromatography (TLC), eluted with CHCl_3_-EtOAc-acetone-acetic acid (2:2:1:0.01, *v*/*v*/*v*/*v*) to provide compounds **1** (3.6 mg), **2** (7.1 mg), **3** (12.3 mg), **4** (5.2 mg), and **5** (1.4 mg).

### 3.3. General Procedure to Synthesize Compounds ***8a*** and ***9a***

In 2.0 mL of mixture of acetic acid and DMSO (3:1, *v*/*v*), methyl β-orsellinate (**8**, 10.0 mg, 0.051 mmol), and sodium bromide (15.76 mg, 0.153 mmol) were dissolved at 80 °C under stirring. Then, 0.5 mL of 30% hydrogen peroxide (4.847 mmol) was added to the reaction flask. The reaction was conducted for 30 min and was periodically monitored every 5 min by TLC. After the reaction mixture was neutralized with saturated sodium hydrogen carbonate, it was further extracted with ethyl acetate-water (1:1, *v*/*v*) to gain an organic layer. The organic layer was washed thoroughly with brine three times, then dried and applied to silica gel column chromatography (CC), eluted with *n*-hexane-EtOAc-acetone (10:1:2, *v*/*v*/*v*) to obtain **8a** (13.3 mg, 95%).

A similar procedure was applied for methyl orsellinate (**9**, 10.0 mg, 0.055 mmol) to obtain product **9a** (16.9 mg, 91%).

### 3.4. General Procedure to Synthesize Compounds ***10a*** and ***10b***

In 5.0 mL of mixture of acetic acid, D-montagnetol (**10**, 50.0 mg, 0.184 mmol) and sodium bromide (56.9 mg, 0.552 mmol) were dissolved at room temperature. Then, 1.8 mL of 30% hydrogen peroxide (17.487 mmol) was added to the reaction mixture. The reaction was conducted for 30 min. The resulting solution was neutralized with saturated sodium hydrogen carbonate, then extracted with ethyl acetate-water (1:1, *v*/*v*) to gain an organic layer. This layer was subsequently washed with brine three times, then dried and applied to silica gel CC, eluted with *n*-hexane-EtOAc-acetone-water (2:2:2:0.01, *v*/*v*/*v*) to obtain **10a** (24.5 mg, 38%) and **10b** (41.6 mg, 53%).

### 3.5. Alpha-Glucosidase Inhibition Assay

*Saccharomyces cerevisiae* α-glucosidase (E.C 3.2.1.20), acarbose, and 4-nitrophenyl *β*-D-glucopyranoside (*p*NPG) were obtained from Sigma Aldrich Co (Saint Louis, MO, USA). The alpha-glucosidase (0.2 U/mL) and substrate (5.0 mM *p*NPG) were dissolved in 100 mM pH 6.9 sodium phosphate buffer [19]. The inhibitor (50 µL) was preincubated with alpha-glucosidase at 37 °C for 20 min, and the substrate (40 µL) was subsequently added to the reaction mixture. The enzymatic reaction was conducted at 37 °C for 20 min and ended by adding 0.2 M Na_2_CO_3_ (130 μL). Enzymatic activity was quantitatively measured at an absorbance of 405 nm (CLARIOstar plus, BMG LABTECH, Ortenberg, Germany). All samples were analyzed in triplicate at five different concentrations around the IC_50_ values, and the mean values were retained. The following equation was used to calculate the inhibition percentage (%): Inhibition (%) = [1 − (A_sample_/A_control_)] × 100.

### 3.6. Antimicrobial Activity Assay

The agar well diffusion method was utilized to investigate the antibacterial activity of the isolated compounds on antibiotic-resistant, pathogenic bacteria *E. faecium*, *S. aureus*, and *A. baumannii*. Three bacterial pathogens were cultured in nutrient broth at 37 °C for 18 h. The cultures were diluted with sterile 0.9% NaCl to obtain bacterial solutions of 1.5 × 10^8^ CFU/mL. This solution with a volume of 100 μL was spread on a Mueller-Hinton agar plate. Holes with a diameter of 8 mm were punched aseptically to create wells on the surface of the Mueller–Hinton agar. The compounds were dissolved in DMSO. The amount of 50 µg of each compound solution was inserted into the wells. The plates were incubated at 37 °C for 16–18 h and the antibacterial activity of each compound was recorded by measuring the diameters of the inhibition zones surrounding the wells. The determination of the minimum inhibitory concentration of **9a** against *Staphylococcus aureus* was performed by the agar dilution method [23]. Compound **9a** was dissolved in DMSO to the final concentration of 1 mg/mL and then diluted with Mueller–Hinton agar (MHA) to the concentration range of 0, 1, 2, 4, 8, 16, 32, 64 μg/mL as the final concentrations in MHA plates. *S. aureus* was cultured in nutrient broth at 37 °C overnight with shaking. The bacterial culture was diluted with sterile 0.9% NaCl to the concentration of 10^7^ CFU/mL. 1 μL of the diluted bacterial solution (10^4^ CFU) was placed on the surface of the MHA plates and the plates were incubated at 37 °C for 16–18 h. The MIC value was recorded as the lowest concentration of **9a** that inhibited the growth of *S. aureus*. Kanamycin was chosen as the positive control in this experiment, while DMSO was regarded as a control.

### 3.7. Molecular Docking Studies

The PDB structures of proteins (4j5t and 1t2p) were downloaded from the Protein Data Bank, while the 3D structures of ligands were modeled via the website chemicalize.com. After the conversion from PDB files into a PDBQT format by AutodockTools, the docking study was designated on AutoDock4.2 using Lamarckian genetic algorithm with 250 runs on the maximum number of evals being 25,000,000 (long) for each ligand–protein complex. The configurations with the most repetitions were employed to extract the estimated free energy as a scoring function for predicting the binding affinities to the macromolecular targets.

### 3.8. Structure Elucidation of the Compounds

Gravity column chromatography was performed on silica gel 60 (0.040–0.063 mm, Merck, Darmstadt, Germany). TLC for checking chromatographic patterns of fractions and isolated compounds was carried out on silica gel 60 F_254_ (Merck, Darmstadt, Germany) and spots were visualized by spraying with 10% H_2_SO_4_ solution followed by heating. Specific rotations were obtained on a Jasco P-1010 polarimeter (Oklahoma City, OK, USA). The HR-ESI-MS were recorded on a MicrOTOF-Q mass spectrometer (Bruker, Billerica, MA, USA). The NMR spectra were measured on a Bruker Avance 500 MHz spectrometer (Bruker, Billerica, MA, USA).

#### 3.8.1. 3,5-Dihydroxybenzoic acid (**1**)

White amourphous powder. ^1^H NMR (500 MHZ, Acetone-*d*_6_) *δ*_H_ 7.03 (*d*, J = 2.0 Hz, 2H, H-2,6), 6.58 (*t*, J = 2.0 Hz, 1H, H-4). ^13^C NMR (125 MHz, Acetone-*d*_6_) *δ*_C_ 167.6 (C-7), 159.4 (C-3,5), 133.4 (C-1), 108.9 (C-2,6), 107.8 (C-4).

#### 3.8.2. 3,5-Dihydroxybenzoate methyl (**2**)

White amourphous powder. ^1^H NMR (500 MHZ, Acetone-*d*_6_) *δ*_H_ 7.00 (*d*, J = 2.0 Hz, 2H, H-2,6), 6.59 (*t*, J = 2.0 Hz, 1H, H-4), 3.83 (*s*, 3H, 7-OMe). ^13^C NMR (125 MHz, Acetone-*d*_6_) *δ*_C_ 167.0 (C-7), 159.5 (C-3,5), 133.1 (C-1), 108.6 (C-2,6), 108.0 (C-4), 52.2 (7-OMe).

#### 3.8.3. 3,5-Dihydroxy-4-methylbenzoic acid (**3**)

White amourphous powder. ^1^H NMR (500 MHZ, Acetone-*d*_6_) *δ*_H_ 7.11 (*s*, 2H, H-2,6), 2.13 (*s*, 3H, 4-Me). ^13^C NMR (125 MHz, Acetone-*d*_6_) *δ*_C_ 167.7 (C-7), 157.0 (C-3,5), 129.4 (C-1), 108.5 (C-2,6), 108.4 (C-4), 8.9 (4-Me) (see Figures S1 and S2).

#### 3.8.4. 3,5-Dihydroxy-4-methoxylbenzoic acid (**4**)

White amourphous powder. ^1^H NMR (500 MHZ, Acetone-*d*_6_) *δ*_H_ 7.10 (*s*, 2H, H-2,6), 3.78 (*s*, 3H, 4-OMe).

#### 3.8.5. 3-Hydroxyorcinol (**5**)

Colorless oil. ^1^H NMR (500 MHZ, Acetone-*d*_6_) *δ*_H_ 6.17 (*s*, 2H, H-2,6), 2.17 (*s*, 3H, 4-Me).

#### 3.8.6. Atranol (**6**)

White amourphous powder. ^1^H NMR (500 MHZ, CDCl_3_) *δ*_H_ 10.34 (*s*, 1H, 3-CHO), 6.29 (*s*, 1H, H-5), 2.53 (*s*, 3H, 6-Me).

#### 3.8.7. Methyl hematommate (**7**)

White amourphous powder. ^1^H NMR (500 MHZ, Acetone-*d*_6_) *δ*_H_ 12.87 (*s*, 1H, 2-OH), 12.41 (*s*, 1H, 4-OH), 10.26 (*s*, 1H, 4-CHO), 6.25 (*s*, 2H, H-2,4), 3.96 (*s*, 3H, 7-OMe), 2.23 (*s*, 3H, 1-Me).

#### 3.8.8. Methyl 5-bromo-β-orsellinate (**8a**)

Isolated yield 95%; White amourphous powder. ^1^H NMR (500 MHZ, Acetone-*d_6_*) *δ*_H_ 11.49 (*s*, 1H, 2-OH), 8.27 (*s*, 1H, 2-OH), 3.95 (*s*, 3H, 7-OMe), 2.61 (*s*, 3H, 6-Me), 2.13 (*s*, 3H, 3-Me). ^13^C NMR (125 MHz, Acetone-*d*_6_) *δ*_C_ 172.4 (C-7), 161.4 (C-2), 156.7 (C-4), 138.4 (C-6), 111.2 (C-3), 107.7 (C-1), 107.3 (C-5), 52.7 (7-OMe), 23.2 (6-Me), 9.3 (3-Me).

#### 3.8.9. Methyl 3,5-dibromo-orsellinate (**9a**)

Isolated yield 91%; White amourphous powder. ^1^H NMR (500 MHZ, Acetone-*d_6_*) *δ*_H_ 11.66 (*s*, 1H, 2-OH), 3.99 (*s*, 3H, 7-OMe), 2.62 (*s*, 3H, 6-Me). ^13^C NMR (125 MHz, Acetone-*d*_6_) *δ*_C_ 171.6 (C-7), 159.3 (C-2), 155.9 (C-4), 140.7 (C-6), 109.5 (C-1), 106.6 (C-5), 97.6 (C-3), 53.3 (7-OMe), 23.2 (6-Me). (see Figures S3 and S4).

#### 3.8.10. 3-Bromo-D-montagnetol (**10a**)

Isolated yield 38%; White amourphous powder. ^1^H NMR (DMSO-*d_6_*) *δ*_H_ 10.45 (br*s*, 1H, 2-OH), 10.02 (s, 1H, 2-OH), 6.42 (*s*, H-5), 4.38 (*dd*, J = 11.0, 2.5 Hz, H-1′a), 4.13 (*dd*, J = 11.0, 6.5 Hz, H-1′b), 3.66 (*m*, 1H, H-2′), 3.56 (*m*, 1H, H-3′), 3.39 (*m*, 2H, H-4′), 2.28 (*s*, 3H, 6-Me). ^13^C NMR (125 MHz, DMSO-*d_6_*) *δ*_C_ 167.8 (C-7), 155.9 (C-2), 155.4 (C-4), 136.8 (C-5), 114.0 (C-1), 102.7 (C-3), 100.9 (C-5), 72.4 (C-2′), 69.3 (C-3′), 67.0 (C-1′), 63.0 (C-4′), 20.7 (6-Me). HRESI-MS *m/z* 348.9929 [M − H]^−^ (Calcd for C_12_H_14_BrO_7_: 348.9923). [α]_D_^25^ +86 (*c* 0.1, MeOH).

#### 3.8.11. 3,5-Dibromo-D-montagnetol (**10b**)

Isolated yield 53.0%; White amourphous powder. ^1^H NMR (DMSO-*d_6_*) *δ*_H_ 10.25 (br*s*, 1H, 2-OH), 4.42 (*dd*, J = 11.0, 2.5 Hz, H-1′a), 4.23 (*dd*, J = 11.0, 6.5 Hz, H-1′b), 3.69 (*m*, 1H, H-2′), 3.57 (*m*, 1H, H-3′), 3.39 (*m*, 2H, H-4′), 2.38 (*s*, 3H, 6-Me). ^13^C NMR (125 MHz, DMSO-*d_6_*) *δ*_C_ 167.4 (C-7), 153.4 (C-2), 153.2 (C-4), 136.4 (C-5), 114.2 (C-1), 105.8 (C-5), 99.0 (C-3), 72.4 (C-2′), 69.0 (C-3′), 67.5 (C-1′), 63.0 (C-4′), 21.3 (6-Me). HRESI-MS *m/z* 426.9041 [M − H]^−^ (Calcd for C_12_H_13_Br_2_O_7_: 426.9028). [α]_D_^25^ +154 (*c* 0.1, MeOH).

## 4. Conclusions

Seven monoaromatic compounds—3,5-dihydroxybenzoic acid (**1**), 3,5-dihydroxybenzoate methyl (**2**), 3,5-dihydroxy-4-methylbenzoic acid (**3**), 3,5-dihydroxy-4-methoxylbenzoic acid (**4**), 3-hydroxyorcinol (**5**), atranol (**6**), and methyl hematommate (**7**)—were isolated and structurally elucidated from the lichen *Parmotrema cristiferum*. Four synthetic compounds—methyl 5-bromo-β-orsellinate (**8a**), methyl 3,5-dibromo-orsellinate (**9a**), 3-bromo-D-montagnetol (**10a**), 3,5-dibromo-D-montagnetol (**10b**)—were synthesized using electrophilic bromination from the starting materials, methyl β-orsellinate (**8**), methyl orsellinate (**9**), and D-montagnetol (**10**). All compounds were evaluated for their alpha-glucosidase inhibition. Compounds **3**, **5**, and **7** showed strong alpha-glucosidase inhibition with IC_50_ values of 24.0, 97.3, and 61.8, respectively. Other compounds showed weak or inactive. Molecular docking studies were performed, providing consistent data between in vitro and in silico studies of **3, 5,** and **7**, thus implying those compounds as potential candidates for further investigation. In addition, compounds were also evaluated for the antimicrobial activity against antibiotic-resistant, pathogenic bacteria *Enterococcus faecium, Staphylococcus aureus*, and *Acinetobacter baumannii*. Only the synthetic compound **9a** exhibited significant activity against *Staphylococcus aureus* with an MIC value of 4 µg/mL. The docking study of **9a** provided the molecular understanding of antimicrobial activity.

## Figures and Tables

**Table 1:** Alpha-glucosidase inhibitory activity of compounds **1**–**10**, **8a**, **9a**, **10a**, and **10b**.

Compound	IC_50_ (µg/mL)
**1**	112.3 ± 0.7
**2**	157.9 ± 2.1
**3**	24.0 ± 0.8
**4**	171.1 ± 2.9
**5**	97.3 ± 1.3
**6**	>300
**7**	61.8 ± 0.4
**8**	>300
**9**	>300
**10**	>300
**8a**	166.7 ± 2.8
**9a**	156.2 ± 2.9
**10a**	133.9 ± 4.5
**10b**	129.5 ± 2.0
Acarbose	317.0 ± 3.1

**Table 2:** Inhibition zones of antimicrobial activity of compounds **1**–**10**, **8a**, **9a**, **10a**, and **10b** (at the concentration of 50 µg/mL).

Compound	Inhibition Zone (mm) 50 µg/mL		
	*Staphylococcus aureus*	*Acinetobacter baumannii*	*Enterococcus faecium*
**1**	-	-	-
**2**	-	-	-
**3**	-	-	-
**4**	-	-	-
**5**	-	-	-
**6**	-	-	-
**7**	-	-	-
**8**	18	16	13
**9**	13	-	-
**10**	-	-	-
**8a**	12	-	-
**9a**	29	-	-
**10a**	-	-	13
**10b**	-	-	-
Apramycin	21	20	21

**Table 3:** The free energy of the complexes.

Compound	Docking (kcal/mol)	Binding Energy Based IC_50_ Values (kcal/mol)	No of H-Bond	Residues	No of Hydrophobic Interactions	Residues
**3-4j5t**	−4.2	−5.3	4	Asn453, Gly566, Trp710, Asp392	2	Trp391
**5-4j5t**	−4.0	−4.3	4	Leu563, Asp568, Trp710	2	Trp391, Arg428
**7-4j5t**	−5.7	−4.8	5	Asp392, Trp391, Gly566, Trp710, Glu771	3	Asp392, Glu771
**Acarbose-4j5t**	−6.65	−5.16	12	Trp391, Asp392, Arg428, Glu429, Asp568, Leu563, Gly566, Glu771	1	Asp568
**9a-1t2p**	−4.9	−6.7	5	Asn114, Thr180, Val168	6	Gln172, Leu169, Ile199, Val168
**Apramycin-1t2p**	−5.89	−5.50	5	Asn 114, Ser116, Arg197, Thr180	1	GLn105

## Data Availability

The data presented in this study are available in Supplementary Material.

## Supplementary Materials

The following are available online at https://www.mdpi.com/article/10.3390/molecules27092871/s1, Figure S1: The ^1^H NMR spectrum of **3**, Figure S2: The ^13^C NMR spectrum of **3**, Figure S3: The ^1^H NMR spectrum of **9a**, Figure S4: The ^13^C NMR spectrum of **9a**.

## Sample Availability

Samples of the compounds are not available from the authors.
