# Supplementary material for: Biological Activities of Lichen-Derived Monoaromatic Compounds
Source: Molecules. 2022 Apr 30;27(9):2871. doi: 10.3390/molecules27092871 (PMC9105517; doi:10.3390/molecules27092871)
Supplement: Supplementary file 1 [file molecules-27-02871-s001.zip › molecules-1665743-supplementary.pdf]

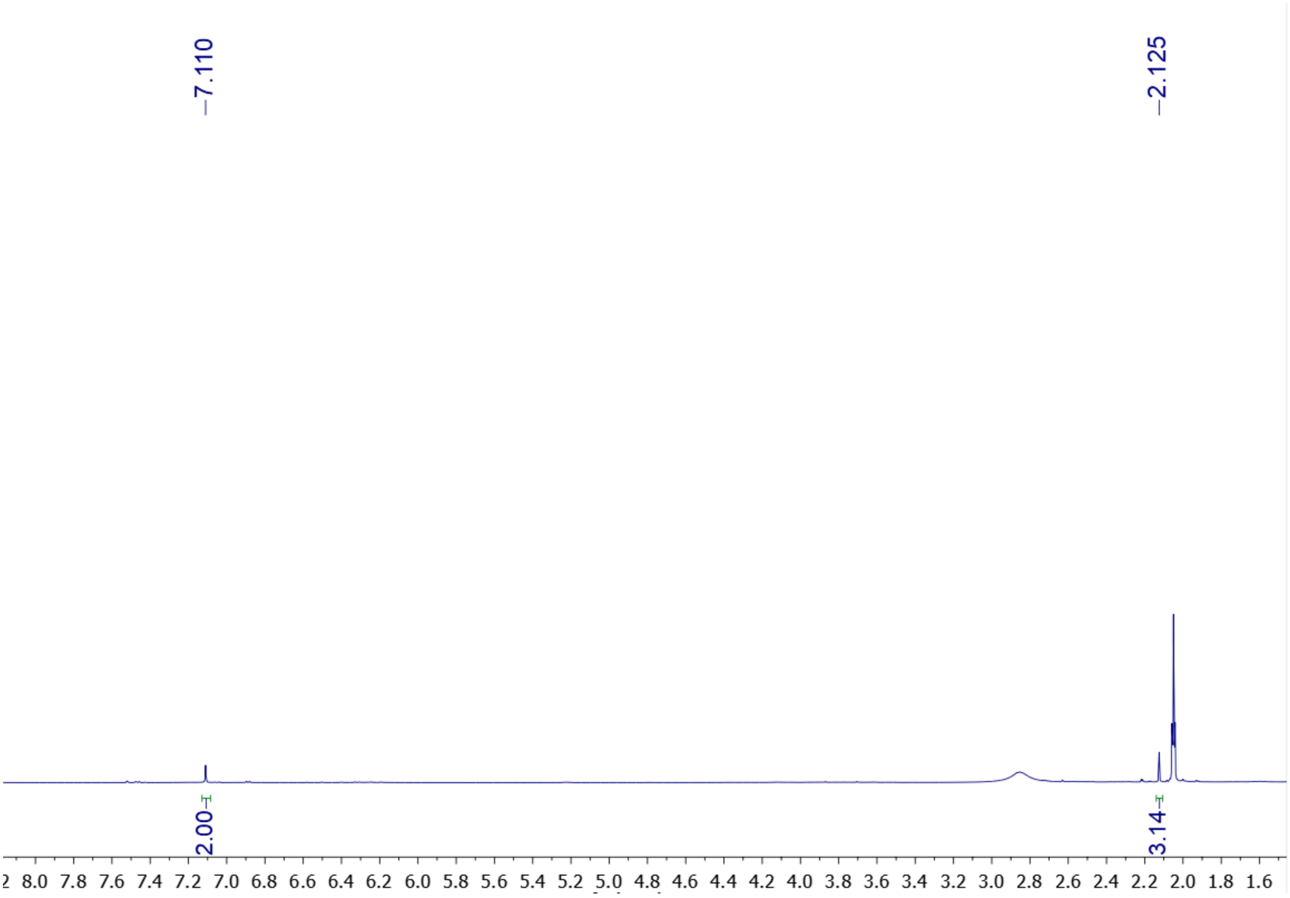

Figure S1. The ^1H NMR spectrum of **3** in acetone-d_6.

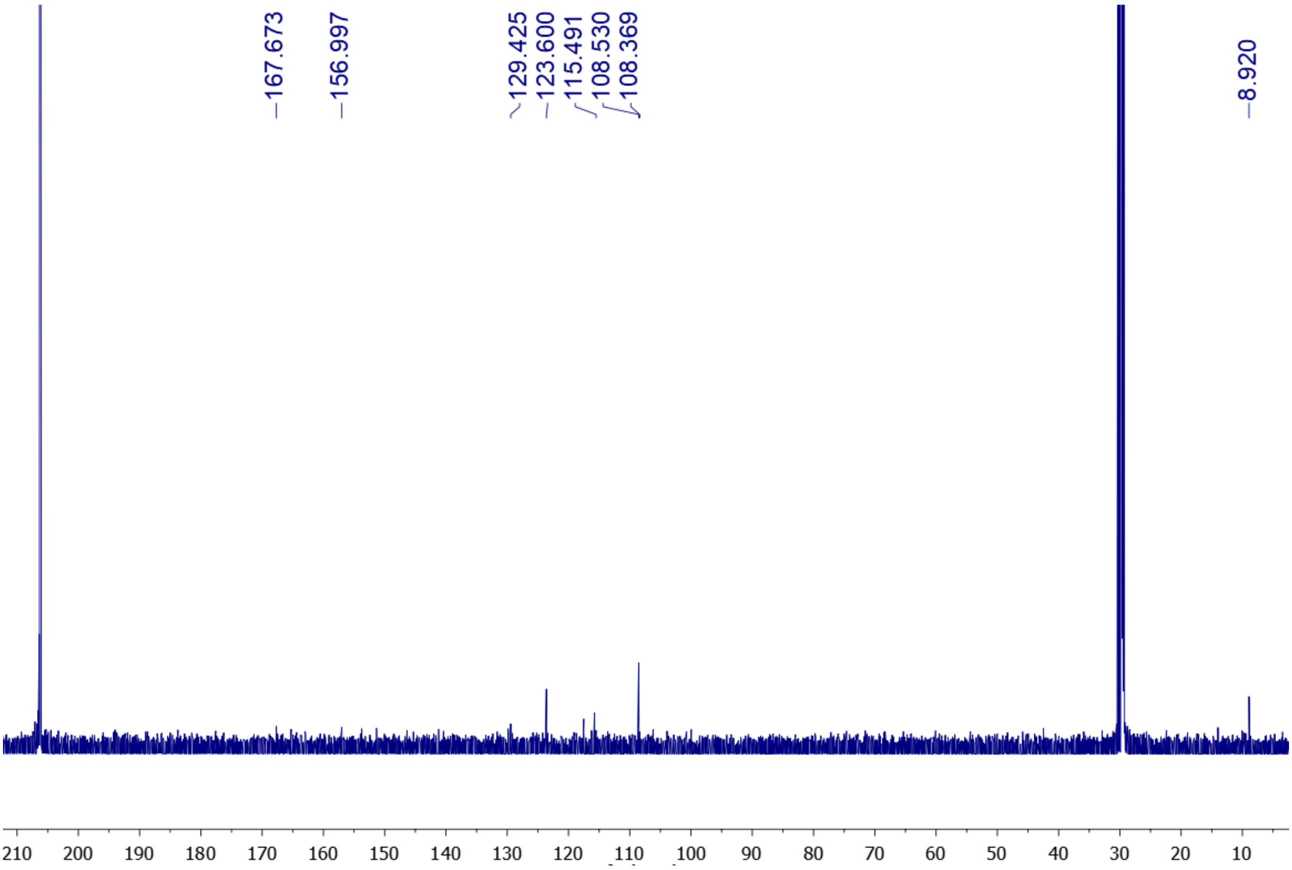

Figure S2. The ^{13}C NMR spectrum of **3** in acetone-d_6.

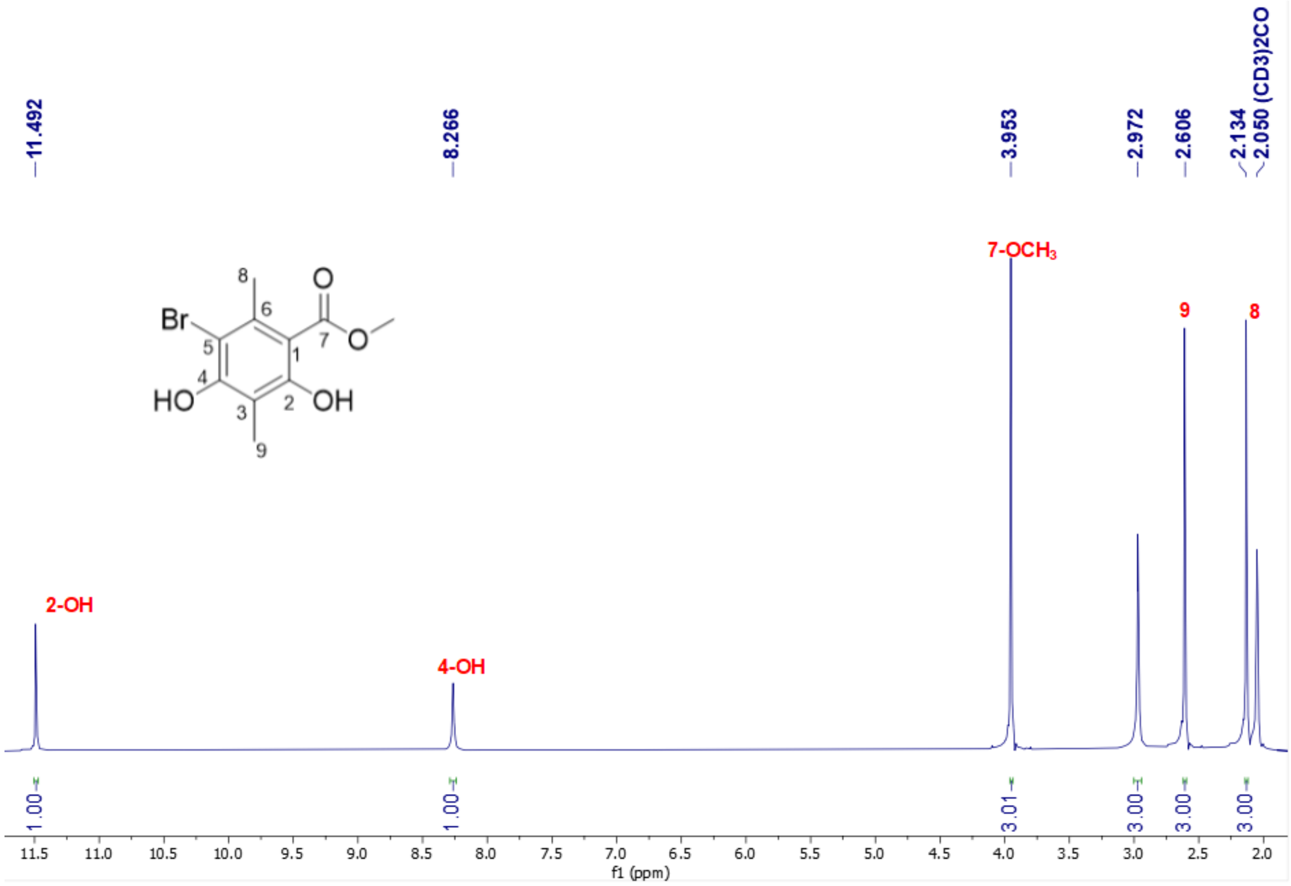

Figure S3. The ^1H NMR spectrum of **9a** in acetone-d_6.

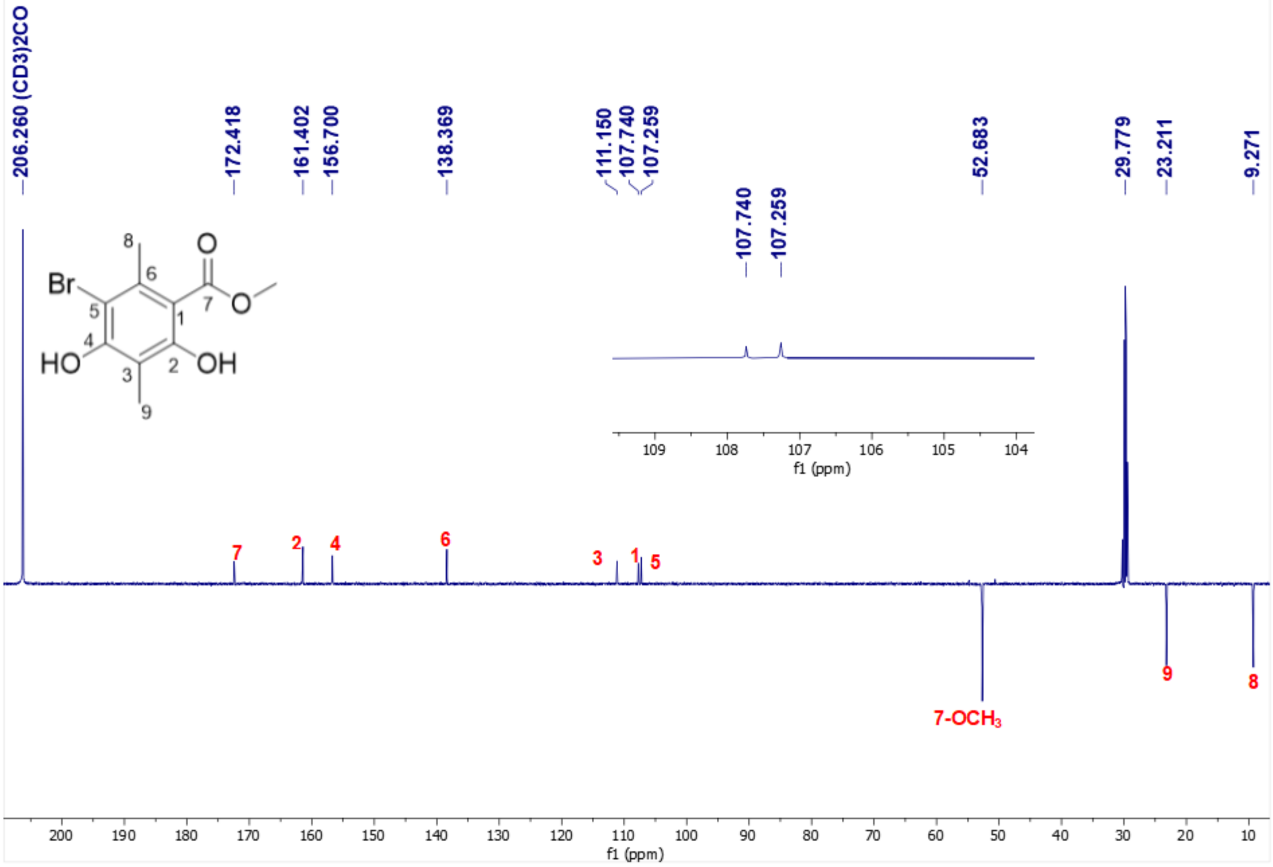

Figure S4. The ^{13}C NMR spectrum of **9a** in acetone-d_6.

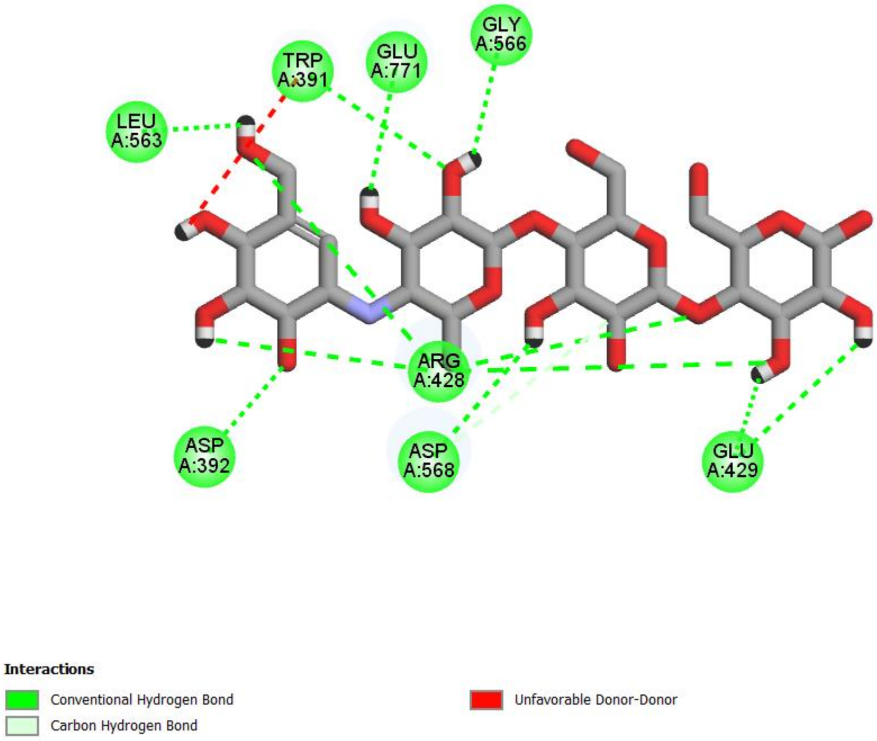

Figure S5. 2D interaction diagram of compound acarbose-4j5t

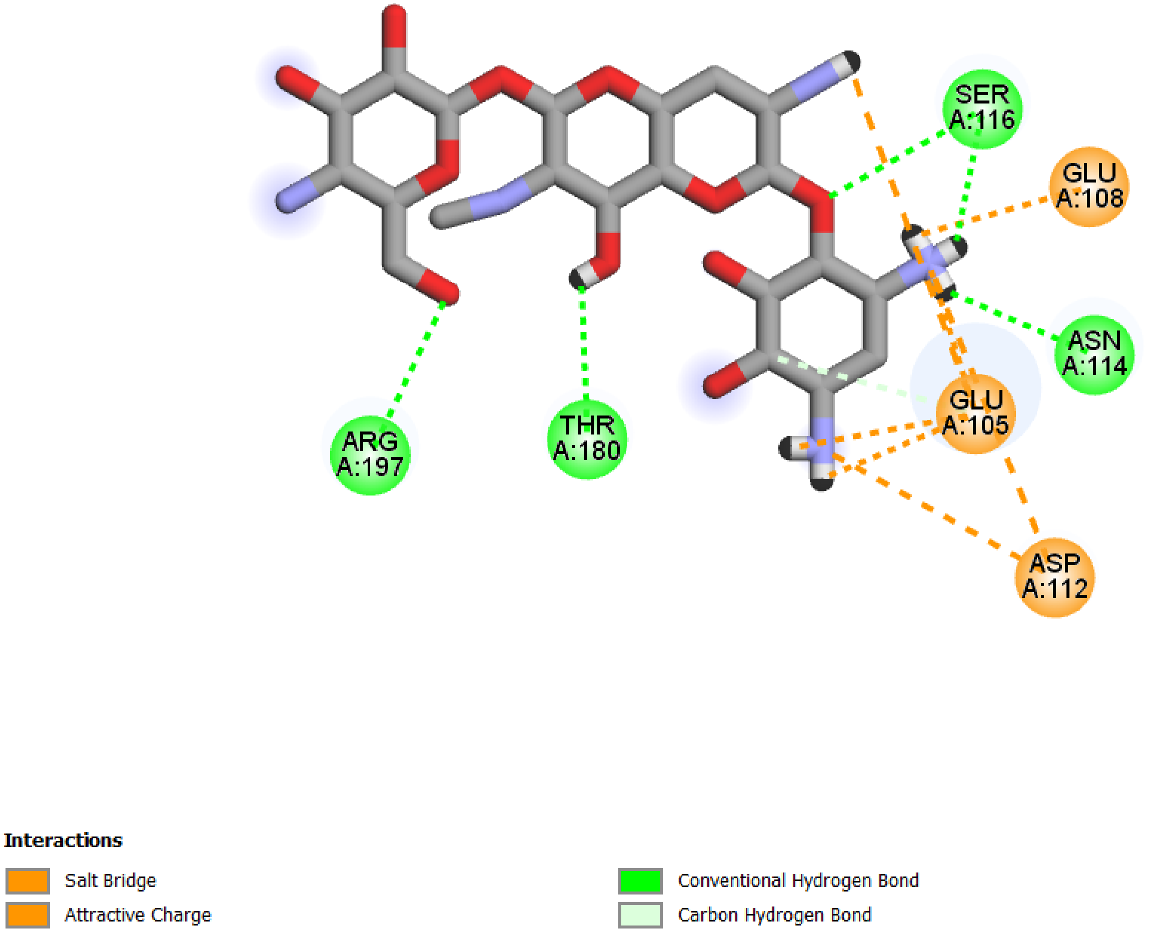

Figure S6. 2D interaction diagram of apramycin-1t2p
